# Microarray analysis of lncRNAs and mRNAs in spinal cord contusion rats with iPSC-derived A2B5^+^ oligodendrocyte precursor cells transplantation

**DOI:** 10.1016/j.heliyon.2023.e22808

**Published:** 2023-11-25

**Authors:** Hao Yuan, Li Chen, Lan-Chun Zhang, Lan-Lan Shi, Xue-Fei Han, Su Liu, Liu-Lin Xiong, Ting-Hua Wang

**Affiliations:** a Department of Neurosurgery, West China Hospital, Sichuan University, Chengdu, 610041, Sichuan, China; b Institute of Neuroscience, Kunming Medical University, Kunming, 650031, Yunnan, China; c Institute of Neurological Disease, West China Hospital, Sichuan University, Chengdu, 610041, Sichuan, China; d Internal Center of Spinal Cord Injury, Johns Hopkins School of Medicine, Baltimore, 21250, Maryland, USA; e Hugo W. Moser Research Institute at Kennedy Krieger Inc., Baltimore, 21250, Maryland, USA; f Department of Anesthesiology, Affiliated Hospital of Zunyi Medical University, Zunyi, 563000, Guizhou, China; g Department of Orthopedics, Affiliated Hospital of Zunyi Medical University, Zunyi, 563000, Guizhou, China

## Abstract

Spinal cord injury (SCI) is a severe complication of spinal trauma with high disability and mortality rates. Effective therapeutic methods to alleviate neurobehavioural deficits in patients with SCI are still lacking. In this study, we established a spinal cord contusion (SCC) model in adult Sprague Dawley rats. Induced pluripotent stem cell-derived A2B5^+^ oligodendrocyte precursor cells (iP-A2B5^+^OPCs) were obtained from mouse embryonic fibroblasts and injected into the lesion sites of SCC rats. Serological testing and magnetic resonance imaging were employed to determine the effect of iP-A2B5^+^OPCs cell therapy. The Basso-Beattie-Bresnahan score and inclined plane test were performed on days 1, 3, 7, and 14 after cell transplantation, respectively. Differentially expressed long non-coding RNAs (lncRNAs) and messenger RNAs (mRNAs) were detected by microarray analysis. Gene Ontology and Kyoto Encyclopedia of Genes and Genomes pathway analyses were performed to analyse the biological functions of these lncRNAs and mRNAs. Real-time quantitative polymerase chain reaction (RT-qPCR) was used to verify variations in the expression of crucial target genes. The results demonstrated that induced pluripotent stem cells exhibited embryonic stem cell-like morphology and could differentiate into diverse neural cells dominated by oligodendrocytes. The neurobehavioural performance of rats treated with iP-A2B5^+^OPCs transplantation was better than that of rats with SCC without cell transplantation. Notably, we found that 22 lncRNAs and 42 mRNAs were concurrently altered after cell transplantation, and the key lncRNA (NR_037671) and target gene (Cntnap5a) were identified in the iP-A2B5^+^OPCs group. Moreover, RT-qPCR revealed that iP-A2B5^+^OPCs transplantation reversed the downregulation of NR_037671 induced by SCC. Our findings indicated that iP-A2B5^+^OPCs transplantation effectively improves neurological function recovery after SCC, and the mechanism might be related to alterations in the expression of lncRNAs and mRNAs, such as NR_037671 and Cntnap5a.

## 1 Introduction

Spinal cord injury (SCI) is an outcome of external violence, usually resulting in high rates of disability and mortality [1]. Approximately 83 people per million suffer from SCI globally, 33 % of these patients lose almost all motor and sensory functions of the hind limbs, and nearly 17 % have quadriplegia [2,3]. Due to its complex pathophysiological mechanisms, there are few effective therapeutic options for improving functional outcomes following SCI [4,5]. Therefore, researchers are increasingly focusing on exploring and developing new therapeutic strategies for SCI.

In recent years, stem cell-based treatments have exerted practical effects in alleviating disorders and restoring injured tissues [6,7][50], especially in studies reporting the efficacy of stem cell transplantation in treating neurological deficits [8,9][49]. A variety of stem cells have been used in the treatment of neurological dysfunction. The most commonly used types include foetal and adult brain stem cells, embryonic stem cells (ESCs), mesenchymal stem cells, and recently popular induced pluripotent stem cells (iPSCs) [10]. IPSCs share common characteristics with the potential of stem cell therapies, which have enriched the stem cell domain and scientific knowledge [11].

IPSCs exhibit characteristics similar to ESCs with pluripotent potential for differentiation [12]. Accumulating evidence demonstrates that iPSC-derived oligodendrocyte precursor cells (iP-OPCs) have been used to treat central nervous system disorders for their contributions to remyelination, axonal regrowth, and functional recovery [13,14]. During oligodendrocyte precursor cells (OPCs) differentiation, new myelin is produced in demyelinated axons via oligodendrocyte formation [15]. Myelin internodes in proliferating OPCs interact with axons through the brain parenchyma, nodes of the nodule, nodules in the nodules, and proximal apical regions [16]. These findings suggest that iP-OPCs transplantation may be an effective therapy for SCI. However, the underlying mechanism requires further investigation.

Long-chain non-coding RNAs (lncRNAs), including sense, antisense, intron, intergenic, and bidirectional lncRNAs, modulate cell status at the transcription levels [17]. LncRNAs are widely recognised as competitive endogenous RNA that were once considered negative substances; however, an increasing number of emerging studies have elucidated their significant roles in regulating cellular activities [18]. LncRNAs variation can contribute to elevating or lowering the levels of specific signalling pathways, thus modifying the fate and function of cells [19]. In a recent study, lncRNAs were found to alter and modify specific protein structures to adjust their stability and prevent functional loss [20]. Several studies indicated that lncRNAs play an indispensable role in SCI repair [21][48]. Hence, the present study was designed to investigate the effect of iPSC-derived A2B5^+^ OPCs (iP-A2B5^+^OPCs) transplantation on the functional recovery of rats with spinal cord contusion (SCC) and to explore the underlying mechanism associated with lncRNAs and mRNAs to clarify the potency of iP-A2B5^+^OPCs transplantation as an effective therapy for SCI.

## 2 Material and methods

### 2.1 Animal care and ethics

**Fig. 1:**
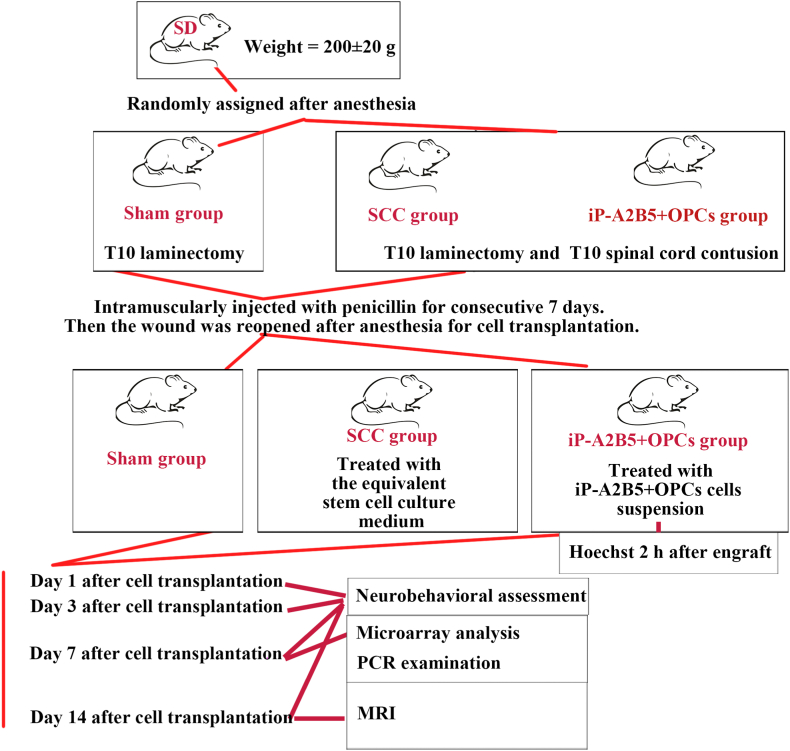
The experimental design and arrangement of this study.

Forty-eight adult female Sprague Dawley (SD) rats weighing 200 ± 20 g, provided by the Laboratory Zoology Department of Kunming Medical University, were randomly divided into three groups using a random number table: sham, SCC, and iP-A2B5^+^OPCs groups (Fig. 1). Detailed information on animal grouping and operations is shown in Table 1. The animals were housed in clean and ventilated conditions with a 12-hour (h) light/dark cycle. Water and food were provided ad libitum. This study was approved by the Experimental Animal Care Committee of the Kunming Medical University, Kunming, China (KMMU2020001). All animal experiments were conducted in accordance with the Institutional Medical Laboratory Animal Management guidelines of Kunming Medical University and were based on the Guide of the United States National Institutes of Health for experimental animal care and safety.

**Table 1:** Animal grouping information and sample size.

Experiments	Time points	Groups	Sample size
Cell engrafting	at 7 d after SCC modeling	iP-A2B5^+^OPCs	n = 18
Hoechst staining	at 2 h after engrafting	iP-A2B5^+^OPCs	n = 3
Microarray	at 7 d after engrafting	Sham	n = 3
		SCC	n = 3
		iP-A2B5^+^OPCs	n = 3
RT-qPCR	at 7 d after engrafting	Sham	n = 6
		SCC	n = 6
		iP-A2B5^+^OPCs	n = 6
Neurobehavior tests	at 1 d, 3 d, 7 d, 14 d after engrafting	Sham	n = 6
		SCC	n = 6
		iP-A2B5^+^OPCs	n = 6
MRI	at 14 d after engrafting	Sham	n = 6
		SCC	n = 6
		iP-A2B5^+^OPCs	n = 6

### 2.2 SCC surgical procedure

The SCC model was established as previously described [22]. All the rats were anaesthetised with an intraperitoneal injection of 3 % pentobarbital sodium (30 mg/kg) and placed in the prone position. The toes of the rats were clamped with a pair of forceps, and the absence of a retraction reaction determined that they were successfully anaesthetised. Then, a 1–1.5 cm incision was made in the middle of the back between the T9 and T11 levels, and the fascia and muscles were separated layer by layer to expose the T10 vertebral laminae. The rats were subjected to T10 laminectomy and then to a 10 g impactor (PA3000; Tektronix, Beaverton, OR, USA) on the indicated spinal cord from a height of 30 mm. Sham rats underwent laminectomies without contusion injury. Subsequently, all animals were intramuscularly injected with penicillin (1 × 10^5^ U/100 g) for 7 consecutive days, and were nursed thrice a day until urination was restored. Vital signs were closely monitored every 2 h after surgery. The day after surgery, the behaviour of the rats was evaluated using the Basso, Beattie, Bresnahan Locomotor (BBB) Rating Scale , and those with BBB scores less than 2 were included in the following experiments.

### 2.3 Production of iPSCs

IPSCs were cultured and differentiated *in vitro* as previously described procedures [23]. Mouse embryonic fibroblasts (MEFs; Global Stem, Gaithersburg, MD, USA) were propagated in Dulbecco's modified Eagle's medium (DMEM; Hyclone, Logan, UT, USA) supplemented with 10 % foetal bovine serum (FBS; Invitrogen, Carlsbad, CA, USA), nonessential amino acids (NEAA; Invitrogen), and penicillin/streptomycin (Invitrogen). The cells were then transfected twice with retroviruses expressing four transcription factors (Oct 3/4, Sox 2, c-Myc, and Klf4) for two days. After four days of culture in DMEM, the transfected cells were subcultured and re-inoculated into MEF pretreated with mitomycin (Invitrogen). After 24 h, the culture agent was replaced with a specific embryonic stem cell culture agent composed of knockout DMEM (Invitrogen), 100 mM nonessential amino acids (NEAA; Invitrogen), 2 mM Glutamax (Invitrogen), 0.1 mM beta-mercaptoethanol (Invitrogen), 1000 U/mL leukaemia inhibitory factor (LIF; Millipore, Billerica, MA, USA), and 20 % serum replacement solution (SR; Invitrogen). IPSCs were subsequently digested with trypsin. On the 16th culturing day, the MEFs were exposed to radiation for amplification.

### 2.4 IPSCs differentiation and iP-A2B5+OPCs identification

The cultured iPSCs were separated from MEFs by 0.25 % trypsin every 4 days until the 3rd passage. Then they were cultivated in the specific stem cell differentiation medium consisting of 50 % DMEM/F12 (Thermo Fisher Scientific, Waltham, MA, USA), 50 % Neurobasal medium (Thermo Fisher Scientific), 1 % N2 (Thermo Fisher Scientific), 1 % B27 (Thermo Fisher Scientific), 10 % serum fluid replacement, 0.1 mM mercaptoethanol (Sigma-Aldrich, Burlington, MA, USA), 2 mM Glutamax (Thermo Fisher Scientific) and 2 μg/mL heparin (Sigma-Aldrich). Gradually, iPSCs cultured in the stem cell medium grew into embryonic bodies. The iPSCs differentiation process lasted 24 days. In addition, primary antibody *anti*-Nestin (ab81462, mouse, 1:100; Abcam) double-labelled with Hoechst (1 μg/mL) was used to observe whether iPSCs clones express characteristic markers of neural stem cells. Finally, the cells were imaged under a fluorescence microscope (Leica AF6000 DMI6000B; Leica Microsystems, Wetzlar, Germany).

The iPSCs were digested into small clumps using Accutase cell digestion liquid at 37 °C for 5 minutes (min), and then the process was terminated by DMEM medium with 2 % foetal bovine serum. Cell count and concentration were determined using 10 μL of the cell suspension. Subsequently, iPSCs were supplemented with A2B5^+^ culture medium composed of 24 mL of 50 % neurobasal medium (Invitrogen), 24 mL of 50 % DMEM/F12 (Invitrogen) with 2 % B27 (Invitrogen), 1 mL of 50 × A2B5^+^ culture medium, 0.5 mL of 100 U/mL penicillin/streptomycin (Thermo Fisher Scientific), 0.5 mL of 100 mM NEAA (Invitrogen), 0.25 mL of 2 mM GlutaMAX (Invitrogen), 0.01 mL of 0.1 mg/mL human fibroblast growth factor (FGF) 2 (PeproTech EC). Subsequently, the cell concentration was adjusted to 1 × 10^5^ cells/mL, and they were inoculated in the incubator containing 5 % CO_2_ at 37 °C. Half of the medium was replaced every other day. Finally, iP-A2B5^+^OPCs were characterised by immunofluorescence staining as described previously [23]. Briefly, cells cultured on glass coverslips were fixed with 4 % paraformaldehyde and blocked with blocking buffer containing 5 % sheep serum and 0.3 % Triton X-100 for 30 min. Afterwards, cells were then incubated with monoclonal primary antibodies at 4 °C overnight: Nestin (ab81462, mouse, 1:200; Abcam), A2B5 (ab53521, mouse, 1:200; Abcam), NG2 (ab129051, mouse, 1:200; Abcam), Olig2 (ab109186, mouse, 1:200; Abcam), PDGFRa (ab96569, mouse, 1:150; Abcam), O4 (LS-C124310, mouse, 1:150; Lifespan Biosciences), NeuN (ab177487, mouse, 1:200; Abcam), NKX2.2 (ab191077, mouse, 1:50; Abcam), GFAP (ab4648, mouse, 1:50; Abcam). After 3 washes, FITC polyclonal antibodies (goat anti-mouse, 1:500; Abnova) were added into plates to incubate at 37 °C for 2 h. After washing three times with PBS, cells were incubated with Hoechst (1 μg/mL) for 5 min at room temperature to visualise the nuclei. Five fields were captured for cell counting using Leica AF6000 DMI6000B (Leica Microsystems).

### 2.5 Cell transplantation

The wound was reopened 7 days after SCC, and the rats were placed on stereotaxis in the prone position. A microinjector containing 5 μL of 1 × 10^5^ cells/mL iP-A2B5^+^OPCs cells suspension was fixed on the stereotaxis and implanted into each rat at two different sites (2.5 mm rostral or caudal to the epicentre of the injured site) at a speed of 2.5 μL/min. The pipette was left in place for 1 min to reduce the efflux of the suspension maximally. The vertebral laminae of rats in the SCC group were reopened and treated with an equivalent volume of iP-A2B5^+^OPCs culture medium. The vertebral laminae of sham rats reopened with neither iP-A2B5^+^OPCs transplantation nor medium treatment. After transplantation, the wound was sutured and the rats were injected with penicillin for 7 days to eliminate haematuria. Additionally, the rats in the medium and cell transplantation groups were subcutaneously treated with 0.05 mL cyclosporine A injection every day for 7 days to suppress immunologic rejection after cell transplantation. Two hours after transplantation, the rats underwent Hoechst labelling to visualise the implanted stem cells *in vivo*.

### 2.6 Basso, Beattie, Bresnahan Locomotor Rating Scale

Motor function of the hind limbs was evaluated using the BBB scoring [24] at 1, 3, 7, and 14 days after cell transplantation. Briefly, rats were placed on a flat and non-slippery bench and their movements were observed for 4 min per BBB rating standards and recorded by three trained researchers blinded to the experimental design. On a 21-point locomotor scale, higher scores indicate better motor function of the hind limbs, with detailed scoring criteria as follows: 0–7 points for joint activity, 8–13 points for gait and coordination function, and 14–21 points for claw movement. Three trials were performed for each rat at intervals of >1 h.

### 2.7 Inclined plane test

An inclined plane test was performed 14 days after cell transplantation. As previously described[47] , the rats were placed on a plane equipped with an indicator of the degree of inclination. The plane was then inclined at a speed of 2°/s, and the degree to which the rat fell from the plane was recorded as the falling degree. Each trial was repeated three times, and the average was recorded.

### 2.8 Magnetic resonance imaging (MRI)

Fourteen days after cell transplantation, MRI scanning was applied to check the spared volume of the injured spinal cord with the 7.0 T MRI scanner (Biospec 70/30; Bruker, Ettlingen, Germany). All rats were anaesthetised by the inhalation of a 2 % isoflurane/oxygen mixture. Next, the rats were fixed in a specific frame for a supine position in the examination bed, and their body temperature was maintained at 37 °C with a heating blanket. T2 Weighted Imaging (T2WI) anatomical images were acquired from the TS with a rapid acquisition using a spin echo (SE) sequence, repetition time (TR) = 3000 ms, echo time (TE) = 12 ms, field of view (FOV) = 3.2 cm × 3.2 cm, matrix = 384 × 384, 25 slices with slice thickness = 0.5 mm, spatial resolution = 0.083 × 0.083 × 0.75. The total acquisition time was 12 min on average 5 times. A microsoftware package was used to convert the DICOM image into the NIFI format. Damage to the spinal cord tissue was shown using iTK-SNAP (http://www.itksnap.org/pmwiki/pmwiki.php).

### 2.9 Tissue and blood collection

Seven days after cell transplantation, the animals were intraperitoneally anaesthetised with pentobarbital sodium (30 mg/kg; Sigma-Aldrich). After successful anaesthesia was confirmed, 1 mL of blood samples from each rat were obtained from the left ventricular arteries for blood biochemical detection. Blood samples were incubated at room temperature for 30 min, followed by centrifugation at 3000 rpm for 5 min. Afterwards, the upper serum was absorbed with a pipette and transferred into a new Eppendorf tube. At last, 300 μL of serum was sampled and detected by the Kuhrman AU480 Blood Biochemical Apparatus. After blood collection, all rats were euthanised under deep anaesthesia with 30 mg/kg 3 % pentobarbital sodium, and death was determined when they did not react to tail clamping. Finally, 0.5 cm spinal cord tissues around the injury centre were harvested and stored in a −80 °C fridge for later microchip detection (n = 3/group) and PCR examination (n = 6/group).

### 2.10 Microarray analysis

2.10

Samples of spinal cord tissue (0.5 cm) around the injury centre were harvested, frozen in a −80 °C fridge, and subsequently transported in dry ice to KangChen Bio-tech Company (Shanghai, China) for lncRNA microchip analysis. Total RNA was extracted using the TRIzol reagent according to the manufacturer's instructions. These were then amplified and transcribed into fluorescent cRNA. The labelled cRNA was then hybridised onto a rat lncRNA Array v2.0 (8–60 K; ArrayStar, Rockville, MD, USA). After washing and fixing, hybridised slides were scanned using an Agilent G2505C microarray scanner (Agilent Technologies, Santa Clara, CA, USA). Finally, the data were analysed using the Agilent Feature Extraction Software (version 11.0.1.1). Differentially expressed lncRNAs and mRNAs among the SCC, sham group and iP-A2B5^+^OPCs group were identified by fold change >2, *p* < 0.05, and the random variance model (RVM) *t*-test, *p* < 0.05.

### 2.11 Gene Ontology (GO) and Kyoto Encyclopedia of genes and genomes (KEGG) analysis

GO enrichment analysis was performed using an online database (www.geneontology.org) to categorise the differentially expressed genes based on biological processes (BP), molecular functions (MF), and cellular components [25]. The KEGG database (www.genome.jp/kegg/) was applied to investigate the enriched pathways of differentially expressed genes.

### 2.12 RT-qPCR

Seven days after cell transplantation, spinal cord tissues from the engrafted cortices were collected and homogenised on ice. Total RNA was extracted using TRIzol reagent (Takara Bio Inc., Otsu, Japan), and the concentration and purity were determined. Total RNA was reverse transcribed into complementary DNA using the Revert Aid First Strand cDNA Synthesis System (Invitrogen). The primer sequences information was as follows: NR_037671, sense, 5′-GTCACGGCTGAGTAAGAAT-3′, antisense, 5′-AACTATACCCAGGCACAAT-3’; Cntnap5a, sense, 5′-TGTTACTGAGGACAAGATTTGG-3′, antisense, 5′-TTCTCAGAGTTGGAACCCT-3’. The amplification was performed using a DNA thermal cycler (ABI 7300; Applied Biosystems, Waltham, MA, USA). β-actin was used as an internal control to evaluate the normalised ΔCt value of each sample. The thermocycling conditions were set as follows: initial denaturation at 94 °C for 5 min, and 35 cycles of amplification at 94 °C for 1 min and 72 °C for 1 min. The relative expression of the targeted gene was normalised to that of β-actin by the 2^─ΔΔCt^ method.

### 2.13 Statistical analysis

Statistical analysis was performed using SPSS 23.0, and all experimental data were displayed as mean ± standard deviation (SD). One-way analysis of variance (ANOVA) with Tukey's post hoc test was performed to detect differences among three or more groups. The Kruskal-Wallis test, followed by Dunn's test, was used to compare BBB scores among the three groups. Statistical significance was set at *p* < 0.05.

## 3 Results

### 3.1 Production of iPSCs

**Fig. 2:**
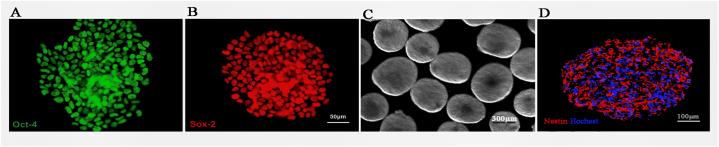
The production of iPSCs. **(A)** IPSCs clones express Oct-4. **(B)** IPSCs clones express Sox-2. **(C)** IPSCs were suspended in stem cell differentiation medium and grew into embryonic bodies. **(D)** IPSCs-derived embryonic bodies surface express Nestin. Scale bar = 50 μm for A and B, 300 μm for C, 100 μm for D.

IPSC clones cultured in stem cell medium showed an embryonic stem cell-like morphology and grew into embryonic bodies (Fig. 2A and B). Afterwards, mouse iPSC-derived embryonic bodies were cultured for 4 days without retinoic acid and purmorphamine, and then for another 4 days with retinoic acid and purmorphamine, generating embryoid bodies with neural progenitors (Fig. 2C). Double-labelled immunostaining results demonstrated that the iPSCs clones expressed the characteristic markers of neural stem cells (Fig. 2D).

### 3.2 Identification and differentiation of iP-A2B5^+^OPCs *in vitro*

**Fig. 3:**
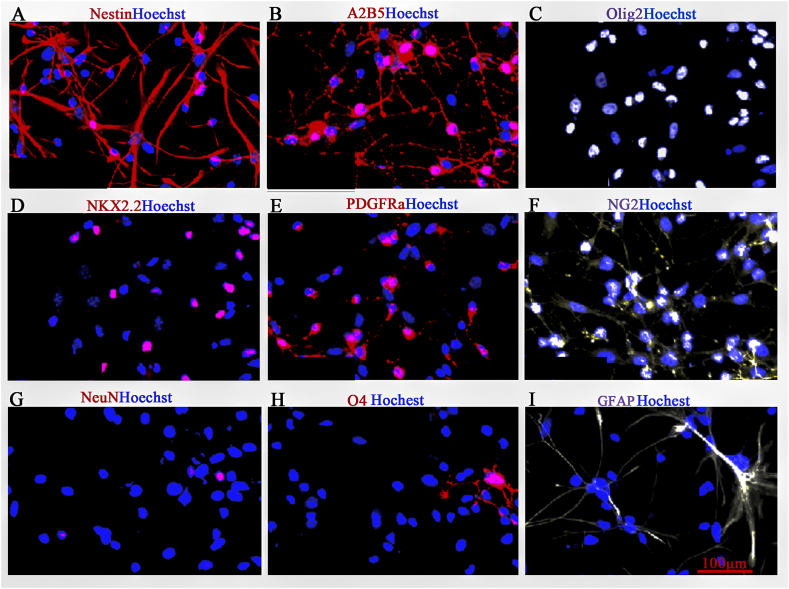
Identification and differentiation of iPSCs *in vitro*. The immunostaining images of **(A)** Nestin positive cells, **(B)** A2B5 positive cells, **(C)** Olig2 positive cells, **(D)** NKX2.2 positive cells, **(E)** PDGFRa positive cells, **(F)** NG2 positive cells, **(G)** NeuN positive cells, **(H)** O4 positive cells, **(I)** GFAP positive cells. Scale bar = 100 μm.

The immunostaining images showed that apparent A2B5^+^ and Nestin^+^ signalling was detected in cultured iP-A2B5^+^OPCs (Fig. 3A and B). The differentiation of iPSC-derived OPCs was identified by PDGFRa, NG2, Olig2, NKX2.2, NeuN, GFAP and O4 immunostaining, which exhibited over 90 % cells expressing Olig2, NKX2.2, PDGFRα and NG2 (Fig. 3C–F), less than 10 % cells expressing O4 and GFAP (Fig. 3H and I), but seldom were NeuN positive cells detected (Fig. 3G). These results demonstrated that iPSCs primarily differentiated into OPCs.

### 3.3 IP-A2B5^+^OPCs transplantation promoted motor function recovery from SCC injury

**Fig. 4:**
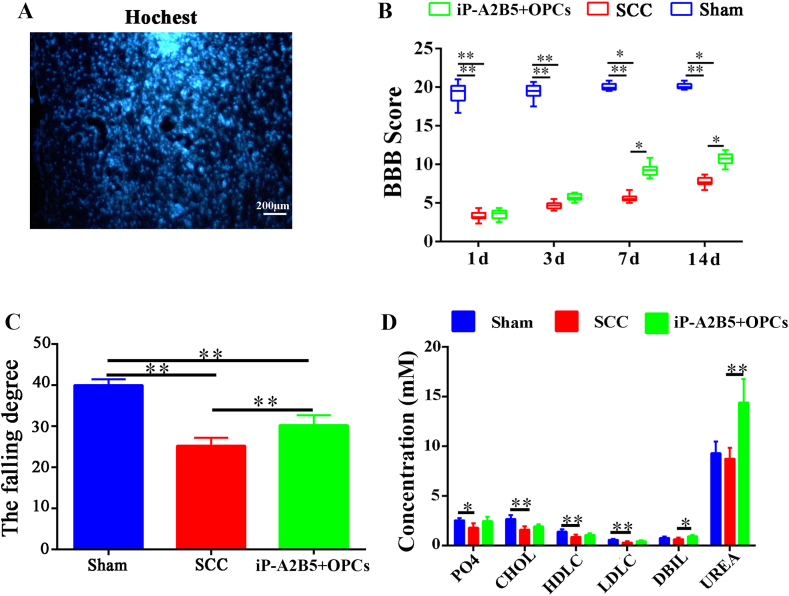
The effect of iP-A2B5^+^OPCs transplantation in SCC rats. (A) The iP-A2B5^+^OPCs in spinal cord tissues were labelled by Hoechst. Scale bar = 200 μm. (B) BBB score evaluation among the sham group, SCC group, iP-A2B5^+^OPCs group at 1, 3, 7, 14 days after cell transplantation. (C)The inclined test was performed at 14 days after cell transplantation. (D) The blood biochemical items, CHOL, HDLC, LDLC, PO4, DBIL and UREA in the serum of rats among sham, SCC and iP-A2B5^+^OPCs group. iP-A2B5^+^OPCs, induced pluripotent stem cells-derived A2B5^+^ oligodendrocyte precursor cells; SCC, spinal cord contusion; CHOL, cholesterol; HDLC, high density lipoprotein cholesterol; LDLC, low density lipoprotein cholesterol; PO4, phosphorus; DBIL, direct bilirubin. Data were expressed as means ± SD. *p < 0.05, **p < 0.01.

After iP-A2B5^+^OPC transplantation, the implanted cells were in good condition (Fig. 4A). One day after cell transplantation, BBB scores of rats in SCC (3.32 ± 0.55) and iP-A2B5^+^OPCs groups (3.53 ± 0.62) were significantly lower compared with that of the sham group (19.23 ± 1.36) (*p* < 0.01, Fig. 4B). With the time prolonged, the limb motor function of rats in SCC and iP-A2B5^+^OPCs groups gradually improved, indicated by BBB scores in the iP-A2B5^+^OPC group (5.72 ± 0.45, 9.27 ± 0.79, 10.72 ± 0.79) were markedly higher than scores in SCC group at 3 days (4.67 ± 0.48), 7 days (5.62 ± 0.46) and 14 days (7.74 ± 0.60) after cell transplantation (*p* < 0.01, Fig. 4B). The scores in the sham group remained much higher than SCC group and iP-A2B5^+^OPCs group within 14 days (19.23 ± 1.36, 19.42 ± 0.96, 20.03 ± 0.44, 20.09 ± 0.39) (*p* < 0.01, Fig. 4B). The inclined plane test revealed that on the 14th day, the maximal falling degree of the rats in the SCC group was significantly lower than that in the sham group (*p* < 0.01), whereas the maximal degree was notably augmented in the iP-A2B5^+^OPCs group (*p* < 0.01, Fig. 4C). Blood biochemical indices were measured to evaluate abnormalities in liver function, kidney function, myocardial enzymes, blood lipids, and blood glucose levels in the animals after cell transplantation. Compared to the sham group, the concentrations of cholesterol (CHOL), high-density lipoprotein cholesterol (HDLC), low-density lipoprotein cholesterol (LDLC), and phosphorus (PO_4_) decreased significantly in the SCC group (*p* < 0.01, *p* < 0.05); however, no significant differences in the four indices were observed between the SCC and iP-A2B5^+^OPCs groups (Fig. 4D). However, the concentrations of direct bilirubin (DBIL) and UREA increased significantly after iP-A2B5^+^OPCs transplantation compared with those in the SCC group (Fig. 4D, *p* < 0.05, *p* < 0.01).

### 3.4 The repair of the injured spinal cords revealed by MRI

**Fig. 5:**
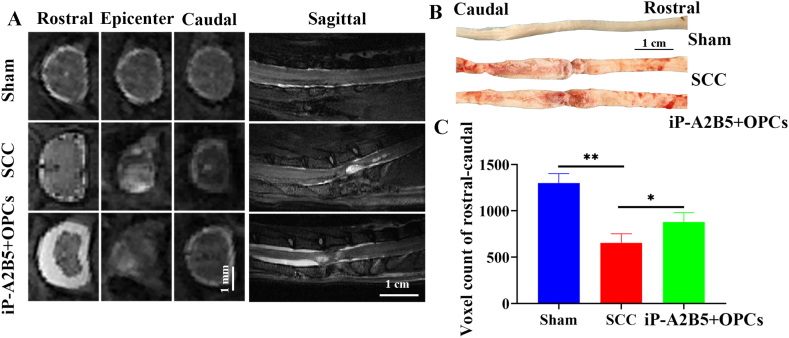
IP-A2B5^+^OPCs transplantation reduced the loss of spinal cord volume. (A) T2 weighted imaging of SCC rats on the 14th day post operation among sham, SCC and iP-A2B5^+^OPCs groups. (B) Macroscopic view of spinal cord tissues in the three groups. (C) Spared volume of injured spinal cord among the three groups. Scale bar = 1 mm for the left 3 columns of panel A, 1 cm for right column of panel A and panel B. iP-A2B5^+^OPCs, induced pluripotent stem cells-derived A2B5^+^ oligodendrocyte precursor cells; SCC, spinal cord contusion. Data were presented as mean ± SD. *p < 0.05, **p < 0.01.

Fourteen days after cell transplantation, we observed the status of the injured spinal cord in the SCC rats using MRI. The spared volume of the spinal cord in the SCC group was markedly reduced compared to that in the sham group (*p* < 0.01, Fig. 5A–C). However, the spared volume of the spinal cord in the iP-A2B5^+^OPCs group was significantly higher (*p* < 0.05, Fig. 5C) than that in the SCC group, indicating that the injured spinal cord was repaired after iP-A2B5^+^OPCs transplantation.

### 3.5 Differentially expressed lncRNAs in spinal cord tissue of SCC rats

**Fig. 6:**
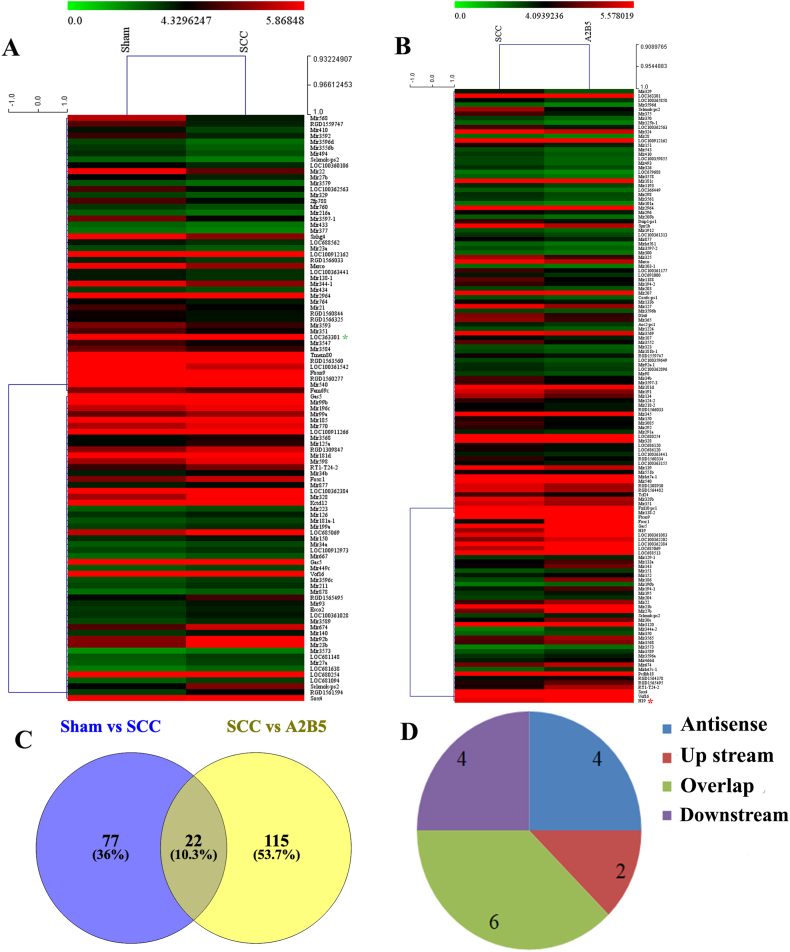
Differentially expressed lncRNAs in the SCC group and iP-A2B5^+^OPCs group. (A) The clustering analysis of differentially expressed lncRNAs between sham group and SCC group. (B) The clustering analysis of differentially expressed lncRNAs between iP-A2B5^+^OPCs group and SCC group. (C) The intersection of differentially expressed lncRNAs in the sham group versus SCC group and SCC group versus iP-A2B5^+^OPCs group. (D) The location relationship of common differentially expressed lncRNAs and their potential target genes. The color intensity of A and B corresponded to the log2 expression values. Red color represents relatively high expression, and blue color represents relatively low expression. LncRNAs, long noncoding RNAs; A2B5 group indicates iP-A2B5^+^OPCs group, induced pluripotent stem cells-derived A2B5^+^ oligodendrocyte precursor cells; SCC, spinal cord contusion. (For interpretation of the references to color in this figure legend, the reader is referred to the Web version of this article.)

Seven days after cell transplantation, the rats were sacrificed for microarray analysis of the changes in mRNAs and lncRNA expression. The variation in lncRNA expression between the sham and SCC groups is shown in Fig. 6A. Among these, 99 differentially expressed lncRNAs were detected in the spinal cord tissue of the SCC group (*p* < 0.05); 59 lncRNAs were upregulated, and 40 were downregulated. The expression of lncRNAs in the SCC and iP-A2B5^+^OPCs groups is shown in Fig. 6B. In total, 137 differentially expressed lncRNAs were identified, of which 97 were upregulated and 40 were downregulated (*p* < 0.05). Of these variant genes, H19 (NR_027324, fold change: 9.496426169337398, *p* < 0.01) was the most upregulated lncRNA, and LOC363301 (ENSRNOT00000041121, fold change: 1.919922, *p* < 0.01) was the most downregulated lncRNA. The combined analysis of the differentially expressed lncRNAs in the three groups revealed that 22 differentially expressed lncRNAs changed concurrently (Fig. 6C and Table 2). After screening the lncRNAs 10 kb upstream and downstream, we found potential target lncRNAs with differential expression, of which there were 2 mRNAs upstream, 4 mRNAs downstream, 6 mRNAs overlapped with lncRNAs, and 4 mRNAs were situated on the antisense strand (Fig. 6D and Table 3).

**Table 2:** Co-changes of lncRNAs in sham-SCC group and A2B5-SCC group.

Regulation	LncRNAs	LncRNAs
down→up	ENSRNOT00000063722	NR_037671
	ENSRNOT00000069622	ENSRNOT00000068851
	ENSRNOT000000628	ENSRNOT00000070304
	ENSRNOT00000063741	ENSRNOT00000062562
	ENSRNOT00000070744	ENSRNOT00000070469
	ENSRNOT00000069185	XR_146025
	ENSRNOT00000054626	
up→down	ENSRNOT00000062394	ENSRNOT00000053486
	ENSRNOT00000069729	ENSRNOT00000062463
	NR_037396	ENSRNOT00000054119
	ENSRNOT00000053156	ENSRNOT00000053182
	ENSRNOT00000069922

**Table 3:** The mRNAs near the co-changed lncRNAs.

LncRNA association	SCC vs sham	A2B5 vs SCC	Gene symbol	Location
ENSRNOT00000062394	up	down	Rab26	antisense
ENSRNOT00000069729	up	down	Ank2	antisense
ENSRNOT00000062463	up	down	Syndig1l	antisense
NR_037396	up	down	Dnm3	antisense
ENSRNOT00000069922	up	down	Fkbp5	Up stream
NR_037671	down	Up	Cntnap5a	Up stream
ENSRNOT00000069622	down	Up	Osbpl5	Overlap
ENSRNOT00000070304	down	Up	Snx25	Overlap
ENSRNOT00000063741	down	Up	Galntl6	Overlap
ENSRNOT00000062562	down	Up	LOC314942	Overlap
ENSRNOT00000053182	down	Up	Kcnab1	Overlap
ENSRNOT00000062365	down	Up	Usp 54	Overlap
ENSRNOT00000070469	down	Up	LOC100364190	Down stream
ENSRNOT00000069185	down	Up	Syn2	Down stream
XR_146025	down	Up	Dpysl5	Down stream
ENSRNOT00000054626	down	Up	Cacng2	Down stream

### 3.6 Differentially expressed mRNAs in spinal cord tissue of rats

**Fig. 7:**
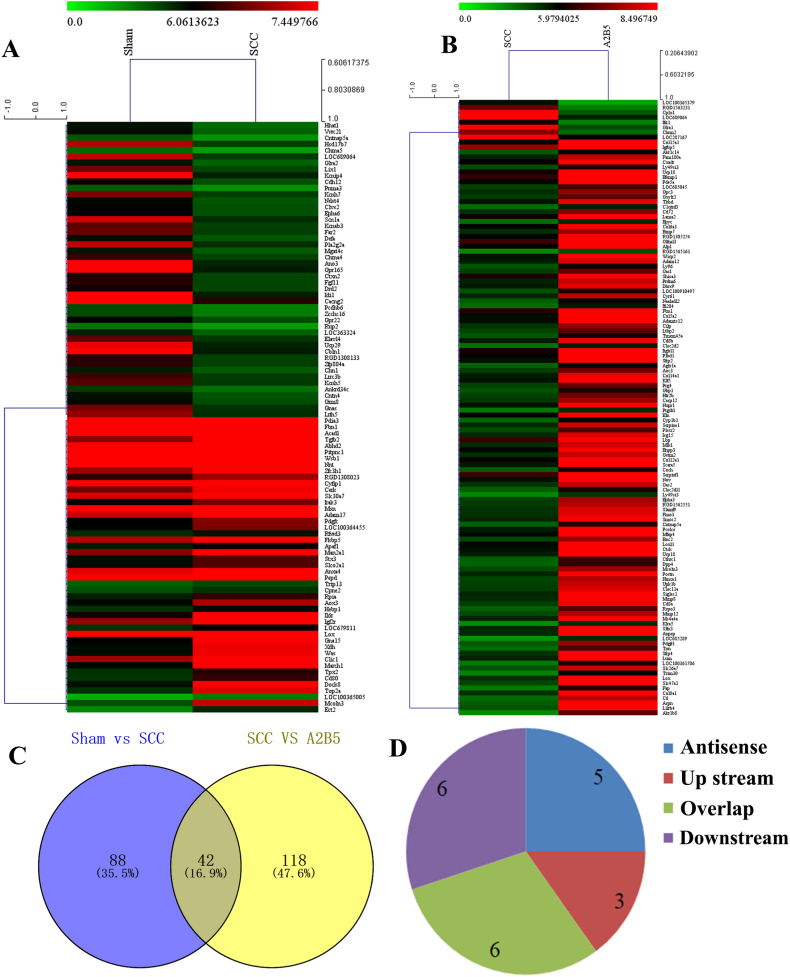
Differentially expressed mRNAs in the SCC group and iP-A2B5^+^OPCs group. (A) The clustering analysis of differentially expressed mRNAs between sham group and SCC group. (B) The clustering analysis of differentially expressed mRNAs between SCC group and iP-A2B5^+^OPCs group. (C) The intersection of differential expression mRNAs in the sham group versus SCC group and SCC group versus iP-A2B5^+^OPCs group. (D) The location relationship of common differentially expressed mRNAs and potential lncRNAs. The color intensity of A and B corresponded to the log2 expression values. Red color represents relatively high expression, and blue color represents relatively low expression. mRNAs, messenger RNAs; A2B5 group indicates iP-A2B5^+^OPCs group, induced pluripotent stem cells-derived A2B5^+^ oligodendrocyte precursor cells; SCC, spinal cord contusion. (For interpretation of the references to color in this figure legend, the reader is referred to the Web version of this article.)

In addition, 130 mRNAs were filtered for differential expression between the sham and SCC group (*p* < 0.05, Fig. 7A and B), of which 47 were upregulated and 83 were downregulated (*p* < 0.05). Moreover, the expression of Mcoln3, Top2a, and Ect 2 was upregulated more than 5-fold. In addition, 160 differentially expressed mRNAs were identified between the iP-A2B5^+^OPCs group and SCC group with a fold change of at least two (*p* < 0.05), of which 126 mRNAs were upregulated and 34 were downregulated (*p* < 0.05). A combined analysis of these mRNAs from the sham group versus the SCC, and SCC groups versus the iP-A2B5^+^OPCs group showed that 42 mRNAs varied concurrently (Fig. 7C and Table 4). Notably, 18 mRNAs were downregulated in the sham group compared to the SCC, but upregulated in the SCC group compared to the iP-A2B5^+^OPCs group. Additionally, two mRNAs were found to be upregulated in the sham group compared to the SCC, but were downregulated in the SCC group compared to the iP-A2B5^+^OPCs group (Table 5). Moreover, three lncRNAs were situated upstream of their mRNAs, six were located downstream, six overlapped with mRNAs, and five were situated in the antisense strand (Fig. 7D and Table 5).

**Table 4:** Co-changes of mRNAs in sham-SCC group versus A2B5-SCC group.

Regulation		mRNAs	
up→down	Cacng2	Kcnip4	Pld5
	Ralyl	Hcn1	
down→up	Elavl2	Rapgef4	Hecw2
	Spock3	Kcnab1	Mdga2
	Necab1	Cacnb4	Tbc1d19
	Sv2b	Acss2	Erbb4
	Slc13a3	Sptb	Dgkg
	Magi2	Stxbp1	Ube2o
	Nrxn3	Srd5a1	Gpc5
	Zfp385 b	Dpysl5	Tfdp2
	Cntnap5a	Grm3	Prkce
	Dlg2	Cxxc4	Grm5
	Lrrc4c	Galnt13	Lrp1b
	Rab11fip4	Syn2	Ass1
	Gpr158

**Table 5:** The lncRNAs near the co-changed mRNAs.

Gene Symbol	SCC vs sham	A2B5 vs SCC	LncRNA Accession	Location
Cacng2	Up	Down	ENSRNOT00000054626	Antisense
Kcnip4	Up	Down	ENSRNOT00000054493	Antisense
Elavl2	down	Up	ENSRNOT00000063135	Antisense
Spock3	down	Up	ENSRNOT00000069157	Antisense
Cacnb4	down	Up	ENSRNOT00000062221	Antisense
Acss2	down	Up	ENSRNOT00000053554	Up stream
Slc13a3	down	Up	ENSRNOT00000053446	Up stream
Sptb	down	Up	ENSRNOT00000070171	Up stream
Magi2	down	Up	ENSRNOT00000069743	Overlap
Stxbp1	down	Up	ENSRNOT00000070381	Overlap
Nrxn3	down	Up	ENSRNOT00000070560	Overlap
Dpysl5	down	Up	XR_146,025	Overlap
Pld5	down	Up	ENSRNOT00000070593	Overlap
Grm3	down	Up	NR_032,260	Overlap
Cntnap5a	down	Up	NR_037,671	Down stream
Dlg2	down	Up	ENSRNOT00000069530	Down stream
Cxxc4	down	Up	ENSRNOT00000063545	Down stream
Lrrc4c	down	Up	ENSRNOT00000070553	Down stream
Syn2	down	Up	ENSRNOT00000069185	Down stream
Gpr158	down	Up	ENSRNOT00000062295	Down stream

### 3.7 GO and KEGG pathway analysis of differentially expressed mRNAs

**Fig. 8:**
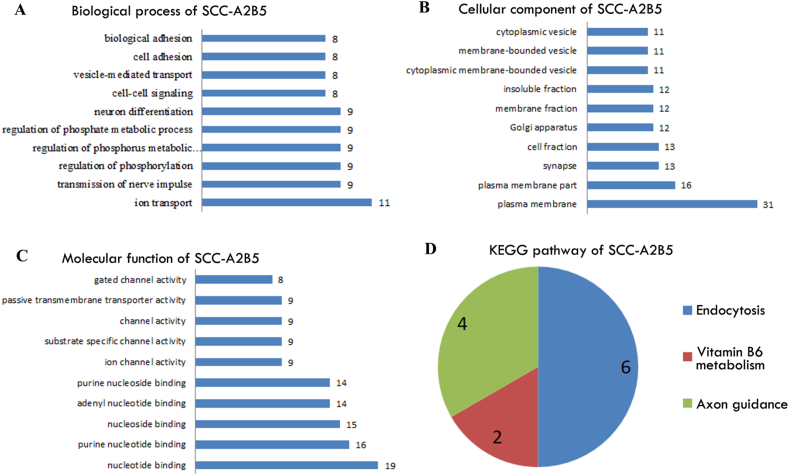
GO and KEGG analysis of differentially expressed mRNAs in the iP-A2B5^+^OPCs group. (A) The top 10 biological processes enriched by differentially expressed mRNAs. (B) The top 10 cellular components enriched by differentially expressed mRNAs. (C) The top 10 molecular functions enriched among differential expression mRNAs. (D) The top 3 pathways enriched by differential expression mRNAs. The bar plot represents the enrichment scores. mRNAs, messenger RNAs; A2B5 group indicates iP-A2B5^+^OPCs group, induced pluripotent stem cells-derived A2B5^+^ oligodendrocyte precursor cells; SCC, spinal cord contusion.

The function of lncRNAs can be recognised by analysing their related mRNAs. Therefore, for special mRNAs with GO enrichment, we can learn about the role of these differentially expressed lncRNAs based on the GO and KEGG enrichment of specific mRNAs. In the iP-A2B5^+^OPCs group, the top ten BP involved in differentially expressed mRNAs were ion transport, transmission of nerve impulse, neuron differentiation, regulation of phosphorylation, regulation of phosphate metabolic process, cell-cell signalling, regulation of phosphorus metabolic process, vesicle-mediated transport, cell adhesion, and biological adhesion (Fig. 8A). The most enriched cell components included the plasma membrane, plasma membrane part, synapse, cell fraction, Golgi apparatus, membrane fraction, insoluble fraction, cytoplasmic membrane-bound vesicle, membrane-bound vesicle, and cytoplasmic vesicle (Fig. 8B). The significantly enriched molecular functions included nucleotide binding, purine nucleotide binding, nucleoside binding, adenyl nucleotide binding, purine nucleoside binding, ion channel activity, substitute specific channel activity, channel activity, passive transmembrane and transporter activity (Fig. 8C). Significant pathways such as endocytosis, vitamin B6 metabolism, and axon guidance were enriched in differentially expressed mRNAs (Fig. 8D).

### 3.8 Co-analysis of mRNAs and lncRNAs between the iP-A2B5^+^OPCs group and SCC group

To obtain the critical lncRNAs involved in the effects of iP-A2B5^+^OPCs transplantation in SCC, we analysed 22 differentially expressed lncRNAs and 42 differentially expressed mRNAs, which were concurrently changed in the iP-A2B5^+^OPCs group versus the SCC group and the sham group versus the SCC group. Potential target genes of the differentially expressed lncRNAs were acquired by scanning 10 kb upstream and downstream. After intersecting the scanned target genes and differentially expressed genes from the microarray, we identified the hub lncRNA Selenok-ps2 (NR_037671) and its target gene (contactin-associated protein-related 5a, Cntnap5a; Table 6). NR_037671 and Cntnap5a were downregulated in the SCC group compared to the sham group (fold change: 2.320 and 2.318, respectively) and upregulated in the iP-A2B5^+^OPCs group compared to the SCC group (fold change: 3.171 and 3.181). Moreover, Cntnap5a is localised downstream of NR_037671.

**Table 6:** The intersected lncRNA and mRNA.

LncRNA association	SCC vs sham	A2B5 vs SCC	Target gene	SCC vs sham	A2B5 vs SCC	Location
NR_037671	Down	Up	Cntnap5a	Down	Up	Down stream

### 3.9 RT-qPCR validation of identified hub lncRNA and mRNA

**Fig. 9:**
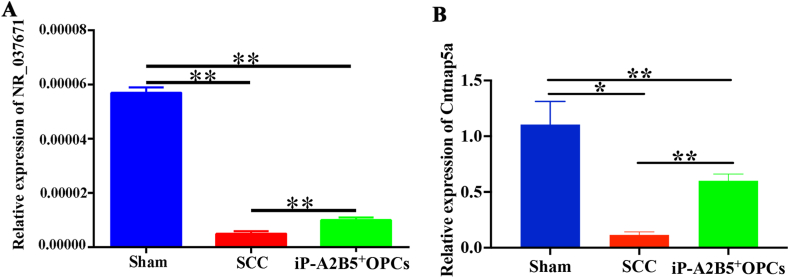
The crucial lncRNA NR_037671 and mRNA Cntnap5a were validated by RT-qPCR. (A) Relative expression of lncRNA NR_037671 in Sham, SCC and iP-A2B5^+^OPCs groups. (B) Relative expression of mRNA Cntnap5a in Sham, SCC and iP-A2B5^+^OPCs groups. LncRNAs, long noncoding RNAs; iP-A2B5^+^OPCs group, induced pluripotent stem cells-derived A2B5^+^ oligodendrocyte precursor cells; SCC, spinal cord contusion. Data were presented as mean ± SD, n = 6/group, *p < 0.05, **p < 0.01.

Finally, RT-qPCR was conducted to verify variations in the expression of NR_037671 and Cntnap5a among the three groups (Fig. 9). Compared to the sham group, the relative expression of NR_037671 and Cntnap5a was significantly lower in the SCC and iP-A2B5^+^OPCs groups (*p* < 0.01, *p* < 0.05), but the levels of NR_037671 and Cntnap5a were elevated in the iP-A2B5^+^OPCs group relative to those in the SCC group (*p* < 0.01, Fig. 9).

## 4 Discussion

In this study, we established an SCC model to explore the effects and potential mechanisms underlying iP-A2B5^+^OPCs transplantation in SCC treatment. Engrafted iP-A2B5^+^OPCs in rats with SCC alleviated spinal cord damage and enhanced neurobehavioural function without obvious side effects. In addition, differentially expressed lncRNAs and mRNAs were determined after cell transplantation, which revealed that the effect of iP-A2B5^+^OPCs transplantation on alleviating SCC injury might correlate with changes in the expression of crucial lncRNAs and mRNAs.

SCI is usually defined as trauma to the spinal cord tissue caused by external mechanical forces and is characterised by motion, sensation, reflexes, and sphincter dysfunction below the plane of damage [26]. The present study adopted the international laboratory general method for spinal cord contusion with strict experimental procedures for establishing an SCC model. After the operation, the rats suffered from double paralysis, valgus of the soles, muscle stiffness, loss of lower limb function, and impaired excretory function, consistent with standard pathological manifestations after spinal cord contusion [27]. This confirmed the successful establishment of SCC animal models in this study. Contusion of the spinal cord causes direct injury to nerve cells and the vascular system, followed by secondary damage to spared nerve cells, destroying neural circuitry [28]. Accordingly, SCC repair approaches have been strategised to replace damaged neural cells during the disease process and induce axonal outgrowth, which fosters myelination to reconstruct neural network links and recover physiological functions [29].

Stem cell treatment allows SCI patients to restore spinal cord function through remyelination and axonal growth to reconnect neural circuits [30]. Numerous stem cell types have a remyelination capacity [31]. OPCs derived from foetal tissues or embryonic stem cells can enhance the regeneration of injured axons, differentiation into neurones, and functional recovery after nervous injury [31,32]. However, extensive ethical concerns and limitations are associated with using embryonic tissues. iPSCs were first generated by Shinya and Yamanaka using somatic cell induction with retroviruses, and their applications have become particularly promising [33,34]. Fortunately, iPSCs derived from MEFs can differentiate into oligodendrocytes [23], mostly overcoming the ethical problems. A2B5, a cell surface ganglioside, is the best marker for developing oligodendroglial progenitors which can facilitate myelin remodelling and suppress axonal degeneration [35]. Therefore, iP-A2B5^+^OPCs were cultured and transplanted into the spinal cords of rats in this study. IPSCs adopt the shape of an embryoid body, a cell aggregate that can efficiently induce the differentiation of iPSCs into specific neural cell types [36]. When cultured in an embryonic stem cell-specialised medium, iPSCs exhibit an embryonic stem cell-like morphology and grow into embryonic bodies. Our results also showed that nestin, a characteristic marker of neural stem cells, was expressed on the surface of the embryonic bodies. The OPCs successfully survived and differentiated into mature oligodendrocytes after transplantation into the injured spinal cord [33]. Consistently, the present study also demonstrated that the iP-A2B5^+^OPCs expressed Oligo 2, Nkx2.2, PDGFRα and NG2, which all are the specific markers of OPCs, and some expressed O4, the marker of mature oligodendrocytes, indicating the iP-A2B5^+^OPCs could differentiate into oligodendrocytes and develop into mature status. Our previous study showed positive MBP expression in OPCs and demonstrated that the implantation of A2B5^+^OPCs promoted myelin formation in SCC rats, revealing that OPCs play an indispensable role in myelinogenesis [37]. These results demonstrated the characteristics of the induced cells and verified the feasibility of iP-A2B5^+^OPCs transplantation for SCC treatment in rats.

After cell transplantation, we used the BBB score and the inclined plane test to evaluate the effect of iP-A2B5^+^OPCs transplantation on neurobehavioural function in SCC rats. The BBB scores in the iP-A2B5^+^OPCs SCC group gradually increased within 3–7 days, and the recovery effect was better than that in the SCC group. Moreover, the inclined-plane test revealed that the neurological function of the iP-A2B5^+^OPC group was better than that of the SCC group. Furthermore, iP-A2B5^+^OPCs transplantation reduced the loss of spinal cord volume compared to that in the SCC group, indicating that iP-A2B5^+^OPCs therapy significantly ameliorated the volume loss of the injured spinal cord. Similar to our previous work, which reported that iP-A2B5^+^OPCs transplantation effectively alleviated neurological dysfunction induced by traumatic brain injury (TBI) [23], the outcomes of this study confirm the neuroprotective role of iP-A2B5^+^OPCs transplantation in SCC. In addition, the potential molecular targets underlying the effect of iP-A2B5^+^OPCs transplantation in TBI were determined using microarray analysis. In contrast to TBI, SCI can result in motion, sensation, reflexes, and sphincter dysfunction below the plane of damage. A previous study has confirmed the protective role of iP-A2B5^+^OPCs transplantation in TBI-induced cognitive and motor deficits in rats. The present study attempted to determine the role of iP-A2B5^+^OPCs transplantation in SCI treatment and explore the preliminary molecular mechanisms, providing a basis for further in-depth mechanistic investigations. Meanwhile, the levels of cholesterol, high-density lipoprotein, and low-density lipoprotein in the serum of rats decreased significantly. However, partial elevation, nearly to the same level as the sham group, showed no statistical difference after cell transplantation, suggesting that iP-A2B5^+^OPCs transplantation can reduce the risk of abnormal lipid metabolism in rats with SCI to some extent. With the increasing incidence of SCI, more patients are being diagnosed with dyslipidaemia using clinical tests [38]. Abnormal blood lipid levels play a crucial role in the pathogenesis of atherosclerosis [39]. IP-A2B5^+^OPCs transplantation can affect dyslipidaemia after SCI, which is beneficial for the recovery of SCI. Recent studies have demonstrated that lncRNAs play a vital role in the differentiation of neural stem cells and the plasticity of synapses [40,41]. Tremendous advances have been made in understanding the mechanisms of lncRNAs, spinal cord pathophysiology, and related diseases [42]. In the present study, microanalysis was employed to evaluate changes in mRNAs and lncRNA expression among iP-A2B5^+^OPCs, SCC, and sham groups. The results showed that improved neurobehavioural dysfunction after iP-A2B5^+^OPCs transplantation may be attributed to variations in the relevant mRNAs and lncRNAs. Synchronous variations were observed in 16 lncRNAs and 42 mRNAs among the three groups.

Specifically, we screened common differentially expressed lncRNAs and mRNAs with a 10 kb range of upstream and downstream range to identify their possible target genes. Ultimately, by intersecting the lncRNA and mRNAs analyses, we obtained the hub lncRNA Selenok-ps2 (NR_037671) and its target gene Cntnap5a (contactin-associated protein-related 5a, also designated as contactin-associated protein 5a, Caspr5a), which is a member of the multidomain transmembrane proteins [43]. Caspr proteins are mainly found in the nervous system, especially at the paranodes and juxtaparanodes, essential components of myelinated axons [44]. Intriguingly, our results revealed decreased mRNA levels of NR_037671 and Cntnap5a in SCC rats, which were reversed by iP-A2B5^+^OPCs transplantation. Therefore, we speculate that NR_037671 and Cntnap5a may be associated with the capacity of iPSCs to differentiate into oligodendrocytes and improve neural function recovery in SCC rats. Moreover, GO and KEGG analyses were employed to explore the biological functions and mechanisms of differentially expressed mRNAs in the iP-A2B5^+^OPCs group. Common differentially expressed mRNAs were mainly related to the protection of the nervous system. Common differentially expressed mRNAs participate in neuronal differentiation. KEGG analysis revealed that the differential genes were mainly enriched in the endocytic, vitamin B6 metabolic, and axon guidance pathways, which are associated with nerve protection and repair [45,46].

## 5 Conclusions

This study confirms the therapeutic efficacy of iP-A2B5^+^OPCs transplantation in accelerating neurological functional recovery from SCC injury. The protective effects of iP-A2B5^+^OPCs transplantation may be attributed to the variations and interactions between the identified differentially expressed lncRNAs and mRNAs.

## Ethical statement

This study was approved by the Institutional Medical Experimental Animal Care Committee of Kunming Medical University (KMMU2020001), and the experiments were conducted under the guidelines for laboratory animal care and safety from the United States National Institutes of Health.

## Funding

This study was financially supported by 10.13039/501100001809National Natural Science Foundation of China (No. 82060243), Zunyi Medical University Future Clinical Doctor Talent Training Plan (No. 2022), Sichuan Province Science and Technology Support Program (2020YFS0043), Key Grant from Yunnan Biological Medicine (No. 2018ZF007-04), and 10.13039/501100008871Applied Basic Research Joint Project of Yunnan Provincial Science and Technology Department and 10.13039/501100003996Kunming Medical University (2019FE001-185).

## Data availability statement

Data associated with our study has not been deposited into a publicly available repository. All data were included in article and supplementary materials referenced in article.

## Additional information

No additional information is available for this paper.

## CRediT authorship contribution statement

**Hao Yuan:** Data curation, Writing – original draft. **Li Chen:** Writing – original draft, Writing – review & editing. **Lan-Chun Zhang:** Formal analysis, Investigation, Visualization. **Lan-Lan Shi:** Formal analysis, Investigation, Methodology. **Xue-Fei Han:** Formal analysis, Investigation, Methodology. **Su Liu:** Formal analysis, Investigation, Methodology. **Liu-Lin Xiong:** Funding acquisition, Writing – review & editing. **Ting-Hua Wang:** Conceptualization, Supervision.

## Declaration of competing interest

The authors declare that they have no known competing financial interests or personal relationships that could have appeared to influence the work reported in this paper.
